# Supplementary figures and images for: Genome-wide identification and expression analysis of ADP-ribosylation factors associated with biotic and abiotic stress in wheat (Triticum aestivum L.)
Source: PeerJ. 2021 Mar 2;9:e10963. doi: 10.7717/peerj.10963 (PMC7934654; doi:10.7717/peerj.10963)

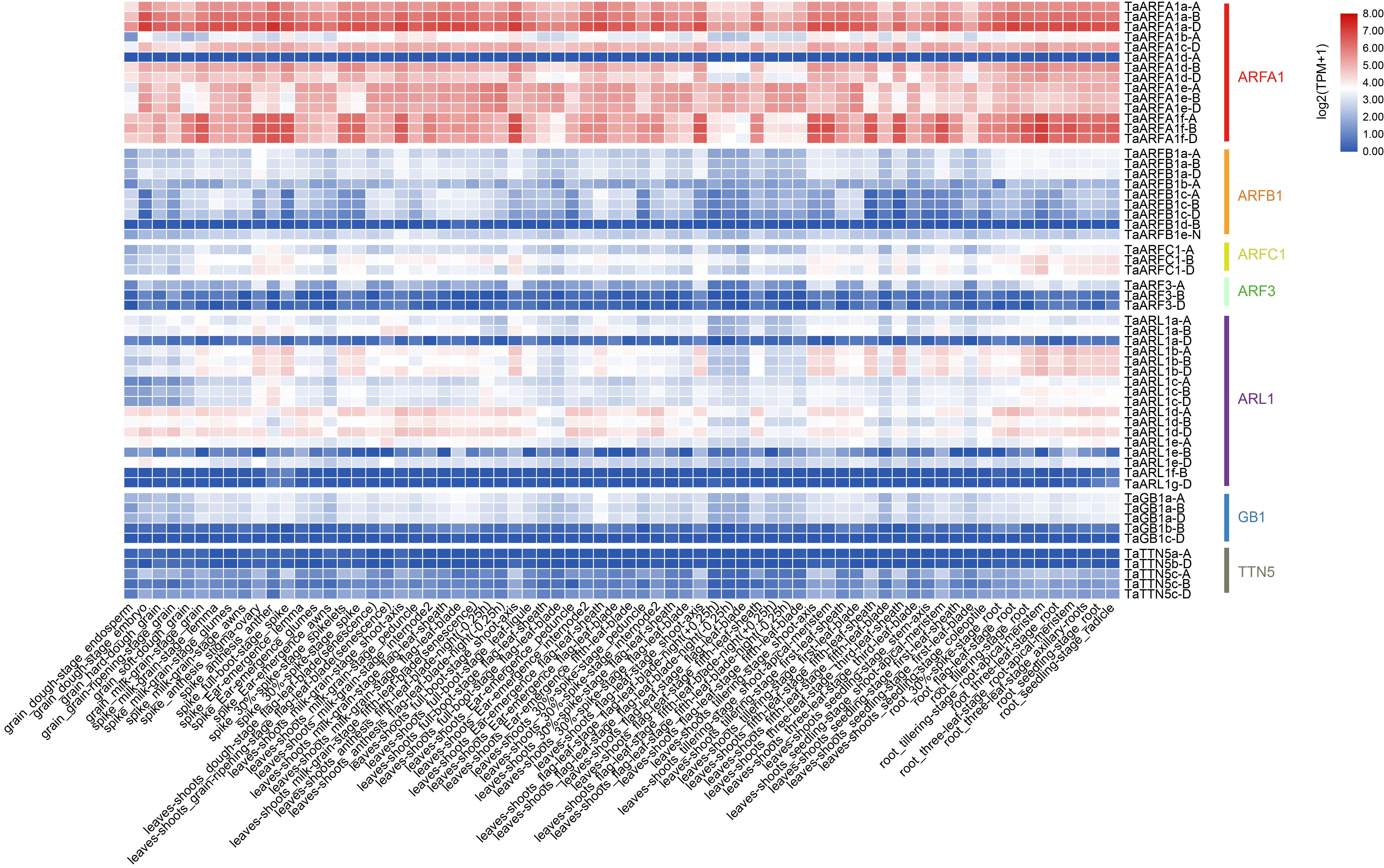

Supplement: Supplemental Information 15 — Expression analysis of all TaARF genes in different tissues, heatmap showing the expression levels of TaARF genes in different subfamilies (columns) and wheat developmental stages/tissues (rows). [file peerj-09-10963-s015.png]

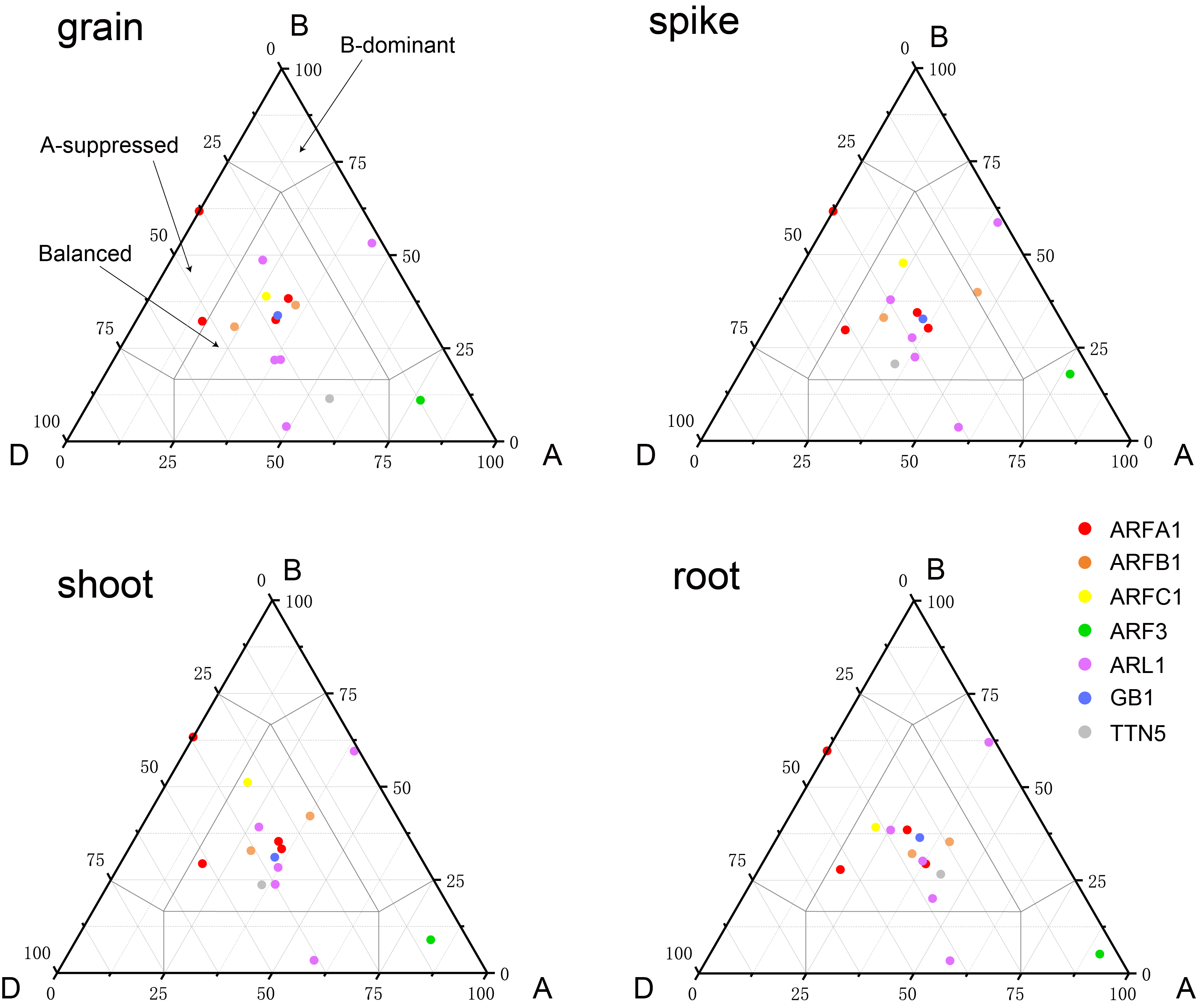

Supplement: Supplemental Information 16 — Expression balance in the grain, spike, shoot and root for all 1:1:1 triads was plotted in a triangular plot with the coordinates of each circle representing the normalized expression of A, B and D homoeologs. Triads are indicated by circles, with areas separated by gray lines indicating expression patterns that are balanced, dominant for one sub-genome homoeolog, or suppressed for one homoeolog, as previously described. Colored circles represent sub-groups. [file peerj-09-10963-s016.png]

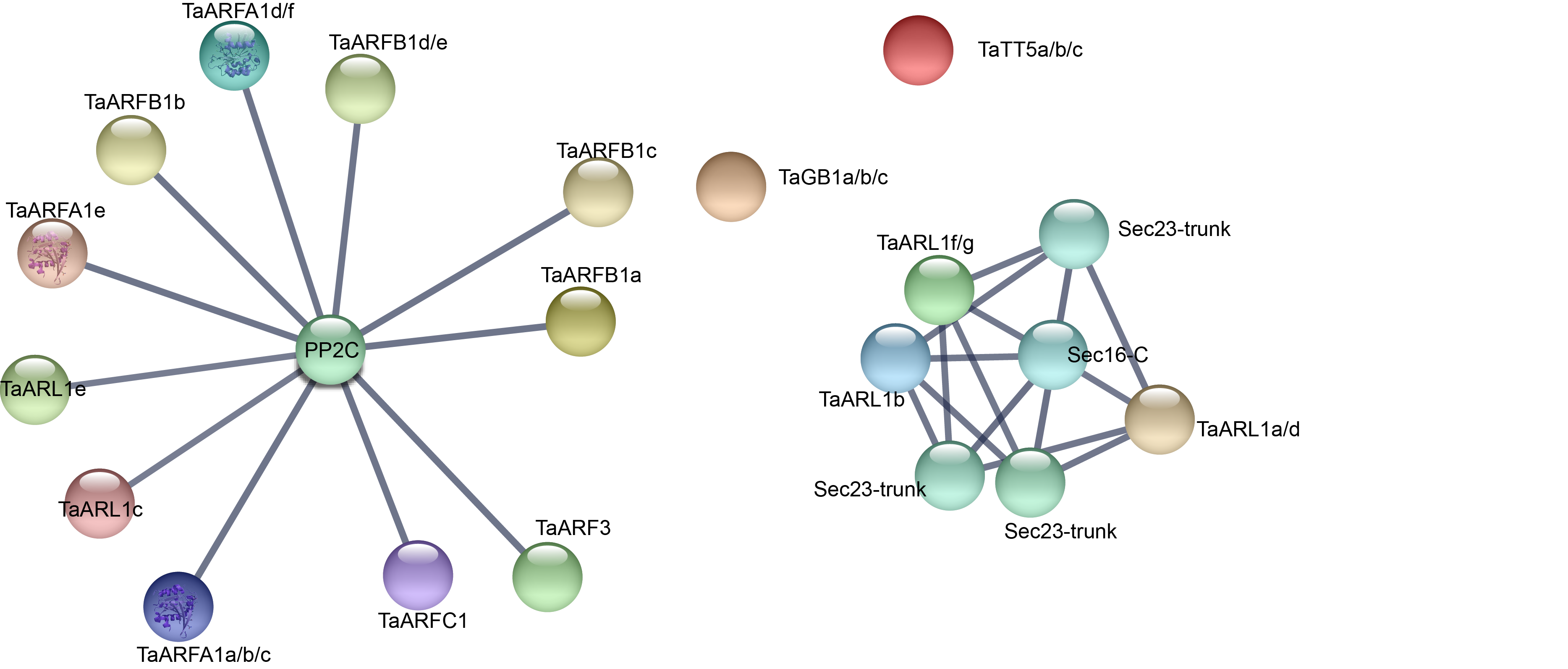

Supplement: Supplemental Information 17 — Expression balance in the grain, spike, shoot and root for all 1:1:1 triads was plotted in a triangular plot with the coordinates of each circle representing the normalized expression of A, B and D homoeologs. Triads are indicated by circles, with areas separated by gray lines indicating expression patterns that are balanced, dominant for one sub-genome homoeolog, or suppressed for one homoeolog, as previously described. Colored circles represent sub-groups. [file peerj-09-10963-s017.png]
